# Relationship between Central Arterial Stiffness and Insulin Resistance in Chinese Community-Dwelling Population without Diabetes Mellitus

**DOI:** 10.1155/2017/1073919

**Published:** 2017-05-14

**Authors:** Shihui Fu, Ying Lin, Leiming Luo, Ping Ye

**Affiliations:** ^1^Department of Geriatric Cardiology, Chinese People's Liberation Army General Hospital, Beijing, China; ^2^Department of Cardiology and Hainan Branch, Chinese People's Liberation Army General Hospital, Beijing, China

## Abstract

**Objective:**

Insulin resistance (IR) is a pathological condition present not only in patients with type 2 diabetes mellitus (DM), but also in community-dwelling population without DM. Both central arterial stiffness and IR are closely correlated with cardiovascular morbidity and mortality. The relationship between central arterial stiffness and IR has not been described in Chinese community-dwelling population without DM. The current analysis was designed to investigate the relationship between central arterial stiffness and IR in Chinese community-dwelling population without DM.

**Methods:**

There were 1150 participants fully assessed for not only homeostasis model assessment of insulin resistance (HOMA-IR) but also carotid-femoral pulse wave velocity (cfPWV).

**Results:**

Median age was 39 (18–80) years, and 69.7% were men. Bivariate correlation analysis showed that cfPWV was significantly related to HOMA-IR (*P* < 0.05). Logistic regression analysis indicated that cfPWV was independently associated with HOMA-IR (*P* < 0.05).

**Conclusions:**

This community-based analysis testified that the relationship between central arterial stiffness and IR was evident as early as during nondiabetic stage. Early interventions in Chinese community-dwelling population without DM to improve the IR are also important in the prevention of cardiovascular diseases.

## 1. Introduction

Insulin resistance (IR) is a pathological condition present not only in patients with type 2 diabetes mellitus (DM) but also in community-dwelling population without DM [1]. IR is closely correlated with cardiovascular morbidity and mortality [2]. The pathophysiological mechanisms underlying these relationships are not well understood but involve abnormal central arterial stiffness. Carotid-femoral pulse wave velocity (cfPWV) is an accepted parameter of central arterial stiffness. It is well known that cfPWV is also related to cardiovascular morbidity and mortality [3, 4].

Homeostasis model assessment of IR (HOMA-IR) is used to assess the degree of IR. Previous studies have analyzed the correlation between central arterial stiffness and HOMA-IR in patients with DM, but the data available on this correlation are lacking and controversial [5]. Some studies have suggested that HOMA-IR is correlated with central arterial stiffness [6, 7], while research has questioned this correlation [8]. Moreover, few studies have involved the correlation between central arterial stiffness and HOMA-IR in population without DM, and there is a need for further studies [9, 10]. In addition, most of these studies have a small sample size and focus on specific age, sex, or race. Due to the scarcity of community-based studies in China, the relationship between central arterial stiffness and IR has not been described thoroughly in Chinese community-dwelling population without DM. To address the above-mentioned problems in China, this analysis sought to investigate the relationship between central arterial stiffness and IR in Chinese community-dwelling population without DM.

## 2. Methods

### 2.1. Study Population

A large health examination survey was carried out in Beijing, China, from May 2007 to July 2009. A stratified cluster sampling design was used in this survey. In the first stage of sampling, three districts (Fengtai, Shijingshan, and Daxing) were selected from 18 districts in Beijing. In the second stage of sampling, four communities were selected from these districts. In the third stage of sampling, participants were selected from these communities. Of 1232 participants older than 18 years (Han origin), 82 participants were excluded because of DM. The final population was 1150 participants.

### 2.2. Anthropometric Indice and Blood Pressure

At the time of initial enrollment, participants underwent a complete physical examination. Well-trained physicians took anthropometric measurements of each participant wearing lightweight clothes and no shoes. Standing height was measured using a wall-mounted measuring tape, and weight was determined with a digital scale. Body mass index (BMI) was calculated as weight divided by height squared. After at least 5 minutes of rest in a sitting position, blood pressure was recorded using a standard sphygmomanometer with the cuff of appropriate size placed on the right arm. Systolic and diastolic blood pressures (SBP and DBP) were from first and fifth Korotkoff phases. Hypertension was defined as SBP ≥ 140 mmHg, DBP ≥ 90 mmHg, or taking antihypertensive drugs.

### 2.3. Glucose Tolerance and Insulin Resistance

Oral glucose tolerance test was conducted between 8 am and 10 am after an overnight fast of at least 12 hours. Participants were given 75 grams of glucose load. Venous blood samples for glucose determination were taken before and 2 hours after glucose load and sent to the central laboratory for analysis within the same day of collection. Blood glucose as well as triglyceride (TG), low-density lipoprotein-cholesterol (LDL-c), and high-density lipoprotein-cholesterol (HDL-c) was checked by qualified technicians blinded to clinical data using the enzymatic assay (Roche Products Ltd, Basel, Switzerland) on a full automatic biochemical autoanalyzer (COBAS 6000; Roche Products Ltd, Basel, Switzerland). DM was diagnosed according to the World Health Organization criteria: fasting blood glucose (FBG) ≥ 7.0 mmol/L, postprandial blood glucose ≥11.1 mmol/L, or taking hypoglycemic drugs. High TG was defined as TG ≥ 1.7 mmol/L. Low HDL-c was defined as HDL-c < 0.9 mmol/L in men or HDL-c < 1.0 mmol/L in women. High LDL-c was defined as LDL-c ≥ 3.37 mmol/L. Fasting insulin (FINS) was determined by DPC kit (DPC cirrus Inc., Los Angeles, CA, USA) on a fully automatic chemiluminescence analyzer (DPC IMMULITE 1000; DPC cirrus Inc.). HOMA-IR was used to assess the degree of IR (HOMA-IR = FINS × FBG/22.5, with insulin in mIU/L and glucose in mmol/L) [11].

### 2.4. Arterial Stiffness and Central Hemodynamics

Automatic cfPWV was assessed using the Complior Colson device (Createch Industrie, Garges les Gonesse, France) in the morning, in a quiet environment, and at a stable temperature; the technical characteristics of this device have been described previously [12]. Participants were studied in the supine position after resting for at least 5 minutes and measured using two strain-gauge transducers [TY-306 Fukuda pressure-sensitive transducer (Fukuda Denshi Co., Tokyo, Japan)] fixed transcutaneously over the course of arteries separated by a known distance; the carotid and femoral arteries (all on the right side) were used. After pulse waveforms of sufficient quality were recorded, the digitization process was initiated by the operator and automatic calculation of the time delay between two upstrokes was started. Measurements were repeated over 10 different cardiac cycles, and mean value was used for the final analysis. cfPWV was calculated from the measurement of pulse transit time and the distance traveled by the pulse between two recording sites (measured on the surface of body in meters), according to the following formula: cfPWV (m/s) = distance (m)/transit time (s).

### 2.5. Statistics

Statistical analyses were performed using Statistic Package for the Social Sciences (SPSS) version 17 (SPSS Inc., Chicago, IL, USA). Categorical data were represented as number and percentage. Continuous data were represented as mean and standard deviation for variables with a normal distribution and median and interquartile range for nonnormally distributed variables. HOMA-IR was a continuous variable with nonnormal distribution and stratified by its median in the current analysis. Statistical comparison was made between groups using Student's *t*-test for continuous variables with a normal distribution, Mann–Whitney *U* test for nonnormally distributed variables, and x^2^ analysis for categorical variables. Pearson or Spearman coefficient was used to assess the bivariate correlation, and then Logistic regression model was constructed to evaluate the multivariate association between HOMA-IR and cfPWV adjusted by age, sex, BMI, hypertension, TG, HDL-c, and LDL-c groups. All statistical analyses were two-sided with *P* < 0.05 as the significant level.

## 3. Results

Median age was 39 (18–80) years, and 69.7% were men. Median HOMA-IR was 1.373 (0.913–2.053), and median cfPWV was 9.6 (8.5–11.0) m/s. Baseline characteristics of the entire population according to HOMA-IR are presented in Table 1. Participants with HOMA-IR > 1.373 had significantly higher values of age, BMI, SBP, DBP, FBG, FINS, PBG, TG, LDL-c, cfPWV, lower value of HDL-c, and higher proportions of BMI ≥ 24, hypertension, high TG, low HDL-c, and high cfPWV than others (all *P* < 0.05). Bivariate correlation analysis showed that age, BMI, SBP, DBP, TG, HDL-c, LDL-c, and cfPWV were significantly related to HOMA-IR (Table 2; all *P* < 0.05). Logistic regression analysis indicated that sex, BMI, high TG, low HDL-c, high LDL-c, and high cfPWV were independently associated with HOMA-IR (Table 3; all *P* < 0.05).

## 4. Discussion

This community-based analysis demonstrated the significant relationship between central arterial stiffness and IR in Chinese community-dwelling population without DM. To our knowledge, this is the first analysis with a community-based design undertaken in Chinese community-dwelling population without DM to explore the relationship between central arterial stiffness and IR.

Previous studies have reported more abnormalities in vascular structure of participants with worsening IR [5]. In line with these studies, this analysis further observed the hardening tendency of arterial system with the deteriorating of IR in Chinese nondiabetic residents. Although the mechanisms linking IR to central arterial stiffness are still unclear, many potential ones have been mentioned. Hyperinsulinemia can increase the sympathetic tone, activate the renin-angiotensin-aldosterone system, stimulate the vascular inflammation, reduce the flow-mediated endothelium-dependent vasodilatation, deplete the nitric oxide or disturb the nitric oxide-mediated vasodilatation, and promote the sodium reabsorption [13–15]. Understanding the relationship between IR and central arterial stiffness as well as the corresponding mechanisms can promote the preventive strategies to reduce the cardiovascular morbidity and mortality.

In summary, this community-based analysis testified that the relationship between central arterial stiffness and IR was evident as early as during nondiabetic stage. This analysis is helpful to understand the relationship between vascular structure and IR. Early interventions in Chinese community-dwelling population without DM to improve the IR are also important in the prevention of cardiovascular diseases.

## Figures and Tables

**Table 1:** Baseline characteristics of the entire population according to insulin resistance.

Characteristics	Low HOMA-IR group (*n* = 575)	High HOMA-IR group (*n* = 575)	*P* value
Age (year)	36 (30–55)	42 (33–59)	<0.001
≤65 (%)	484 (84.2)	472 (82.1)	0.345
>65 (%)	91 (15.8)	103 (17.9)	
Males (%)	405 (70.4)	396 (68.9)	0.564
BMI (kg/m^2^)	23.51 (21.53–25.47)	25.95 (24.22–27.92)	<0.001
<24 (%)	332 (57.7)	131 (22.8)	<0.001
≥24 (%)	243 (42.3)	444 (77.2)	
Hypertension (%)	107 (18.6)	170 (29.6)	<0.001
SBP (mmHg)	120 (111–131)	126 (116–137)	<0.001
DBP (mmHg)	75 (69–81)	79 (71–86)	<0.001
FBG (mmol/L)	4.67 (4.40–4.92)	4.97 (4.68–5.24)	<0.001
FINS (mIU/L)	4.39 (3.21–5.41)	9.39 (7.62–12.3)	<0.001
PBG (mmol/L)	5.11 (4.36–6.02)	5.77 (4.76–6.85)	<0.001
High TG (%)	112 (19.5)	228 (39.7)	<0.001
TG (mmol/L)	1.06 (0.81–1.54)	1.53 (1.13–2.17)	<0.001
Low HDL-c (%)	16 (2.8)	55 (9.6)	<0.001
HDL-c (mmol/L)	1.44 (1.22–1.68)	1.24 (1.06–1.50)	<0.001
High LDL-c (%)	71 (12.3)	70 (12.2)	0.928
LDL-c (mmol/L)	2.40 (1.96–2.92)	2.54 (2.09–3.01)	0.005
High cfPWV (%)	254 (44.2)	324 (56.3)	<0.001
cfPWV (m/s)	9.2 (8.4–10.7)	9.9 (8.7–11.3)	<0.001

HOMA-IR: homeostasis model assessment of insulin resistance; BMI: body mass index; SBP: systolic blood pressure; DBP: diastolic blood pressure; FBG: fasting blood glucose; FINS: fasting insulin; PBG: postprandial blood glucose; TG: triglycerides; HDL-c: high-density lipoprotein-cholesterol; LDL-c: low-density lipoprotein-cholesterol; cfPWV: carotid-femoral pulse wave velocity.

**Table 2:** Bivariate correlation between arterial stiffness and insulin resistance.

Characteristics	HOMA-IR	
	*r*	*P* value
Age (year)	0.120	<0.001
BMI (kg/m^2^)	0.459	<0.001
SBP (mmHg)	0.174	<0.001
DBP (mmHg)	0.200	<0.001
TG (mmol/L)	0.392	<0.001
HDL-c (mmol/L)	−0.305	<0.001
LDL-c (mmol/L)	0.907	0.001
cfPWV (m/s)	0.115	<0.001

HOMA-IR: homeostasis model assessment of insulin resistance; BMI: body mass index; SBP: systolic blood pressure; DBP: diastolic blood pressure; TG: triglycerides; HDL-c: high-density lipoprotein-cholesterol; LDL-c: low-density lipoprotein-cholesterol; cfPWV: carotid-femoral pulse wave velocity.

**Table 3:** Multivariate association between arterial stiffness and insulin resistance.

Characteristics	High/low HOMA-IR group	
	HR (95% CI)	*P* value
Age > 65/≤65 group	0.900 (0.607–1.335)	0.601
Male/female group	0.594 (0.443–0.796)	0.001
BMI ≥ 24/<24 group	4.228 (3.198–5.590)	<0.001
Hypertension/nonhypertension group	1.174 (0.841–1.639)	0.345
High/low TG group	1.836 (1.362–2.476)	<0.001
Low/high HDL-c group	1.453 (1.068–1.977)	0.017
High/low LDL-c group	0.661 (0.440–0.991)	0.045
High/low cfPWV group	1.371 (1.036–1.816)	0.027

HOMA-IR: homeostasis model assessment of insulin resistance; HR: hazard ratio; CI: confidence interval; BMI: body mass index; TG: triglycerides; HDL-c: high-density lipoprotein-cholesterol; LDL-c: low-density lipoprotein-cholesterol; cfPWV: carotid-femoral pulse wave velocity.
